# Unusual cause of intestinal obstruction—Gossypiboma mimicking acute appendicitis in a post-hysterectomy patient: A case report

**DOI:** 10.1016/j.ijscr.2025.111163

**Published:** 2025-03-18

**Authors:** Samrat Shrestha, Kaushal S. Thapa, Mecklina Shrestha

**Affiliations:** a National Academy of Medical Sciences, NAMS, Bir Hospital, Department of General Surgery, Kathmandu, Province-3, Nepal; b College of Medical Sciences (CoMS), Department of Emergency Medicine, Bharatpur, Kathmandu, Province-3, Nepal

**Keywords:** Gossypiboma, Appendicitis, Intestinal obstruction, Retained surgical gauze, Case report

## Abstract

**Introduction and importance:**

Gossypiboma refers to the inadvertent retention of cotton materials, such as gauze, sponges, or towels, inside the body following a surgical procedure. It is most frequently encountered in intra-abdominal or pelvic surgeries, and while preventable, it remains a significant risk.

**Clinical discussion:**

We present a case of a 44-year-old female who underwent total abdominal hysterectomy (TAH) a year ago and presented with right lower abdominal pain, distension, and vomiting. Ultrasonography suggested acute appendicitis and intestinal obstruction, prompting an open appendectomy. During surgery, a retained surgical gauze was discovered 25 cm proximal to the ileocecal junction. The gauze was removed via enterotomy, and the enterotomy was repaired. The patient had an uneventful postoperative recovery.

**Discussion:**

Gossypiboma often occurs in abdominal or pelvic surgeries, with risk factors such as emergency surgeries, high BMI, and excessive bleeding. Literature shows the incidence from 1 in 1000 to 1 in 1500 abdominal operations. It can lead to foreign body reactions like encapsulation, abscess formation, or transmural migration through the intestine. Symptoms can be delayed, and complications may include obstruction or perforation. Diagnosis is challenging but can be aided by imaging like CT scans. Prevention is the key but retained surgical sponges require surgical removal.

**Conclusion:**

Gossypiboma, though uncommon, poses a significant risk after abdominal surgeries and can present with vague, nonspecific symptoms, delaying diagnosis. Strict adherence to surgical protocols, including accurate sponge counts and careful intraoperative monitoring, can help prevent the occurrence of retained foreign bodies and their subsequent complications.

## Highlights

• Gossypiboma refers to a retained surgical gauze or textile material left inside the body after surgery.
• Commonly occurs after abdominal/pelvic surgeries; risk factors: emergency surgery, high BMI, and insufficient sponge counts.
• Diagnosis can be challenging, but CT scans are useful for confirming retained foreign bodies.
• Preventive measures like meticulous surgical techniques and accurate sponge counts, can help reduce the occurrence of gossypiboma.
• Retained surgical sponges require surgical removal.

## 1 Introduction

Gossypiboma refers to a mass of cotton material, typically gauze, sponges, or towels, unintentionally left inside the body during or after a surgical procedure [1]. The term “gossypiboma” is derived from two sources: the Latin word “gossypium,” meaning cotton or textile, and the Swahili word “boma,” meaning a place of concealment [2]. It is also known by other terms, including textiloma and gauzoma. While various surgical materials like artery forceps, broken instrument pieces, irrigation sets, scissors, needles, and rubber items may also be inadvertently left in the body, textile materials are the most forgotten [3]. Gossypiboma can occur after almost any surgical procedure, including thoracic, orthopedic, spinal, neurological, and breast surgeries. However, it is most frequently seen following intra-abdominal or pelvic surgeries [1]. Certain factors increase the likelihood of gossypiboma, including emergency surgeries, changes in surgical plans, patients with high body mass indexes, insufficient attention to sponge counts, and situations with excessive bleeding where gauze swabs are used to control hemorrhage [4]. Although gossypiboma is preventable, eliminating the risk is unlikely [5]. We are reporting a similar case of a 44-year-old female with retained surgical gauze inside the abdominal cavity with subsequent transmigration of surgical gauze inside the ileum. This case has been reported according to the revised SCARE guidelines, 2023 [6].

## 2 Case report

**Fig. 1:**
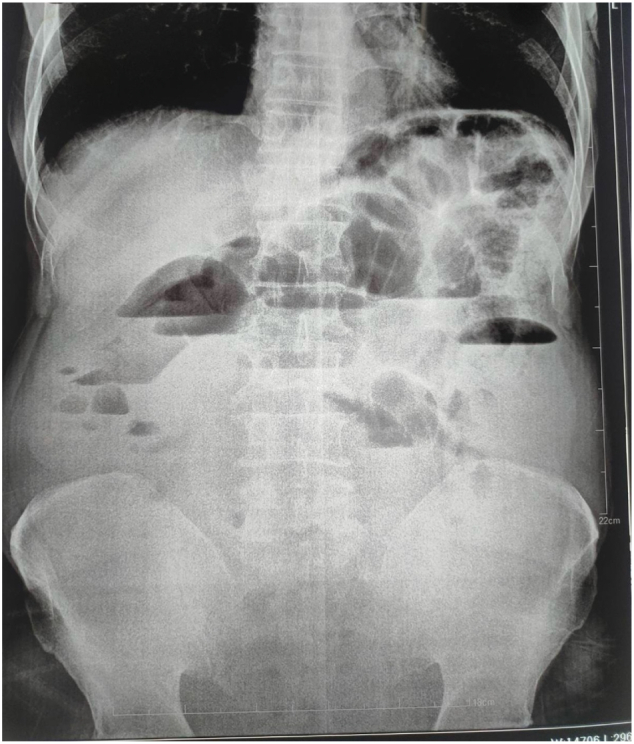
The X-ray abdomen erect view shows multiple air-fluid levels suggesting intestinal obstruction.

**Fig. 2:**
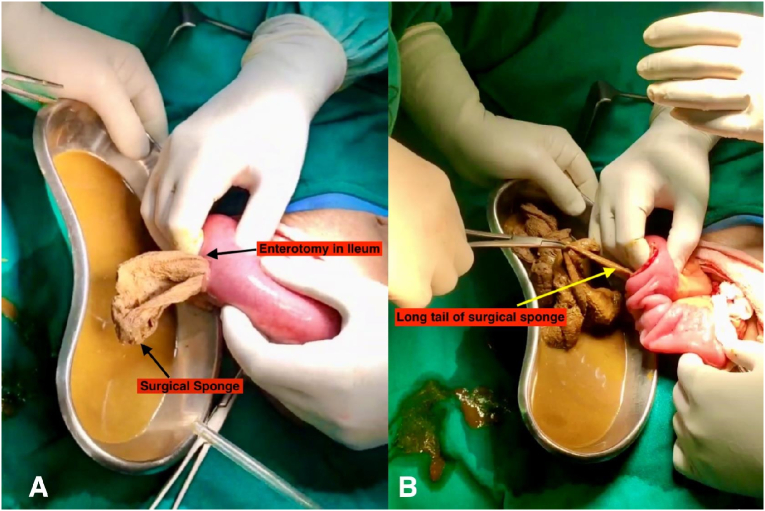
Intraoperative picture shows A: Surgical sponge being extracted from the enterotomy site at the ileum. B: A long tail of a surgical sponge is being pulled out from the enterotomy site at the ileum.

**Fig. 3:**
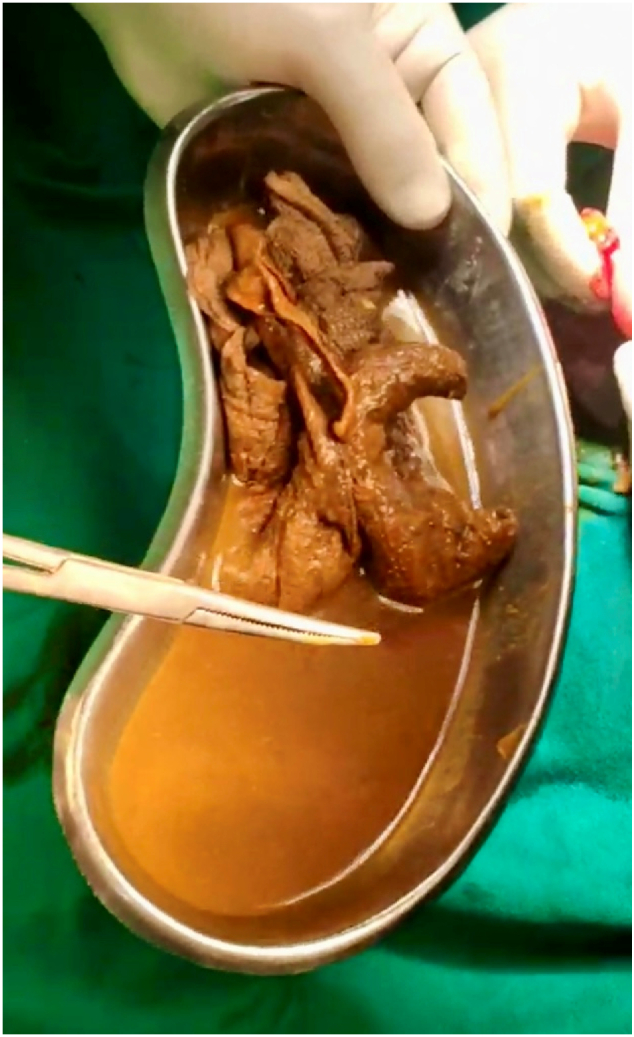
Surgical sponge specimen after being extracted from the ileum.

A 44-year-old female presented to the emergency department of our hospital with a history of right lower abdominal pain for 3 days associated with abdominal distention, a few episodes of vomiting, and anorexia. The pain was not associated with fever. The patient had a history of total abdominal hysterectomy (TAH) done 1 year back at a local hospital for abnormal uterine bleeding (AUB). The patient had no other significant medical comorbidities. She did not have a history of any psychiatric illness. On physical examination, her body mass index was 23.5 kg/m^2^, and her vitals were stable. The abdomen was distended with a Pfannenstiel scar present at the lower abdomen with tenderness over the right iliac fossa; rebound tenderness was present; however, there was no guarding or rigidity. Bowel sound was present but sluggish. On digital rectal examination, the rectum was empty, and no mass was felt. The rest of the physical examination was unremarkable. On blood investigation, hemoglobin was 8.5 g/dl, total leukocyte count was 11,000/cumm with neutrophils of 70 % and lymphocytes of 25 %. The rest of the blood investigations were within normal limits. The modified Alvarado score was calculated as 7/9. Ultrasonography of the abdomen showed features suggestive of acute appendicitis with an appendicular diameter of 8 mm with minimal peri-appendiceal collection. The X-ray abdomen erect view demonstrated multiple air-fluid levels suggestive of intestinal obstruction (Fig. 1). With the preoperative diagnosis of acute appendicitis, open appendectomy was planned by Gridiron incision. The operative finding was an inflamed appendix with a healthy base. There was 50 ml of serous peri-appendiceal collection. The ileum was dilated with a doughy consistency. With suspicion of gossypiboma, an enterotomy was performed, and 30 cm × 30 cm long-tailed surgical gauze was present (Fig. 2, Fig. 3), about 25 cm proximal to the ileocecal junction, which was retrieved through the enterotomy. Enterotomy was repaired in two layers with an interrupted absorbable suture. Post-operative recovery was uneventful; an oral diet was started on the 2nd postoperative day, and the patient was discharged on the 6th postoperative day. On retrospective history taking, the patient denies ingestion of foreign bodies, and there was no history of pica in the past. On the 14th day's follow-up, the patient had no issues, and the wound was healing well. On 1-month, 3-month, and 6-month follow-ups, the patient showed no symptoms or signs of intestinal obstruction.

## 3 Discussion

Gossypiboma can occur after almost any surgical procedure, including thoracic, orthopedic, spinal, neurological, and breast surgeries. However, it is most frequently seen following intra-abdominal or pelvic surgeries [1]. Certain factors increase the likelihood of gossypiboma, including emergency surgeries, changes in surgical plans, patients with high body mass indexes, insufficient attention to sponge counts, and situations with excessive bleeding where gauze swabs are used to control hemorrhage [4]. An article published in 2020 reported 94 cases of intraluminal gossypiboma dating back to 1963 CE. The mean age of the patients was 40.4 years, and females were affected more than males [7]. Obstetric and gynecological surgeries were the leading operative surgeries [7,8]. The term “gossypiboma” refers to a sponge or piece of gauze that is inadvertently left in the surgical field during surgery [7]. Literature shows the incidence from 1 in 1000 to 1 in 1500 abdominal operations [9]. However, these numbers may be less than the actual incidence as the surgeon may not report these cases for fear of litigation [10]. In patients with retained surgical gauze, two types of foreign body reactions can occur. The common reaction consists of an aseptic response resulting in adhesion, encapsulation, and granuloma formation. Patients are asymptomatic, which is detected incidentally or with pseudotumor syndrome. An exudative inflammatory reaction is another foreign body reaction to retained sponges with abscess formation or chronic internal and external fistula. Transmural migration, which occurs rarely, involves the gauze migrating through the intestinal wall and becoming embedded within the lumen of the bowel. The intestine is the most common site for this phenomenon [8]. The animal study done by Wattanasirichaigoon proposed four stages in the process of transmural migration of a surgical sponge. Stage 1 involves a foreign body reaction. The sponge is walled off by the omentum, and some loops of jejunum and ileum participate in encapsulating it. When the sponge contacts the viscera, some areas become ischemic and inflamed and stimulate lytic enzymes, which dissolve tissue in front of it. Stage 2 is secondary infection. When cotton filaments reach the intestinal lumen and contact the various intestinal enzymes, cytolysis occurs. Stage 3 is the stage of mass formation. The infection spreads throughout the sponge, and a thick, fibrous wall protects the host from spillage of infected contents into the peritoneal cavity. A mass is formed by dilated loops of jejunum or ileum; the sponge is within the mass, and the lumen of the mass is in continuity with the lumen of the bowel. Cotton filaments are released into the lumen. In stage 4 remodeling occurs. A fibrotic scar is formed after the whole surgical sponge is released into the lumen [8,10]. The symptoms of gossypiboma are often non-specific and may take weeks, months, or even years to manifest after the initial surgery [11]. As a result, diagnosis can be delayed, potentially leading to significant morbidity or even mortality [12]. The presentation may vary according to location and presents with various clinical features. Transmural migration of retained gauze can cause symptoms of obstruction, as seen in this case, and can also lead to perforation or other complications such as fistula formation. Very few cases of intravisceral migration of retained gauze have been reported [[13], [14], [15]]. Patients with a history of abdominal surgery who later present with symptoms such as abdominal pain, nausea, vomiting, or signs of intestinal obstruction or malabsorption should be suspected of having an abdominal gossypiboma rarely with possible transmural migration [3]. Rarely, fistulas, perforation, or spontaneous expulsion from the anus have also been reported [9,13,15]. Various risk factors have been identified to be associated with retained foreign bodies in surgical procedures. Of the eight risk factors—emergency surgery, unexpected change in procedure, multiple surgical teams involved, nursing staff change during procedure, body mass index (BMI), volume of blood loss, female sex, and surgical counts—three major risk variables—BMI, an unforeseen modification in the procedure, and emergency surgery—were found by multivariate logistic regression to be statistically significant [16]. It is extremely difficult to diagnose transvisceral migration of gauze by clinical examination. X-rays and ultrasounds can help diagnose the condition if the gauze is radio-opaque. However, computed tomography (CT) scans, when available, offer a more definitive diagnosis. CT findings may include a spongiform pattern with gas bubbles or a whorled appearance, which are pathognomonic for retained surgical sponges [1,11,17]. In this case, the diagnostic challenge arose from the patient's presentation that mimicked acute appendicitis, a more common cause of right lower abdominal pain. However, certain clinical features, such as abdominal distension, a history of recent abdominal surgery (TAH), and a sluggish bowel sound, raised the possibility of an underlying foreign body causing obstruction. Clinicians should have a high index of Suspicion and gossypiboma should be considered in patients with a history of abdominal surgery presenting with nonspecific abdominal symptoms, especially if imaging findings are inconclusive or atypical [1,4,12]. Surgeon should have intraoperative vigilance during re-exploration for unexplained abdominal symptoms, as gossypiboma can mimic other conditions like appendicitis or intestinal obstruction [3,9,13]. In our case, a CT was not done before surgery as a clinical diagnosis of acute appendicitis was made. While prevention is key, the only effective treatment for a retained surgical swab in the abdomen is surgical removal, which carries a 10 % mortality risk if delayed. Prevention involves thorough counts of surgical items at various stages of the procedure. The Association of Registered Nurses recommends counting before starting, when adding new items, before closing cavities, and before skin closure, with any discrepancies requiring a full search or a radiograph. The American College of Surgeons also stresses the importance of maintaining an optimal operating environment for focused performance. All surgical sponges and gauzes should contain radio-opaque markers to facilitate detection on imaging if inadvertently retained [1,5,17]. Effective communication among surgical teams, especially during emergencies or changes in surgical plans, can reduce the risk of retained foreign bodies [4,16]. Although alternative methods like percutaneous extraction have been suggested, these are not very effective, especially because laparotomy can reveal dense adhesions between the foreign body and abdominal organs [13,14]. The use of barcoded sponges and radiofrequency identification (RFID) systems can further enhance the accuracy of sponge counts and reduce the incidence of gossypiboma [16,17]. This case highlights the importance of considering gossypiboma in the differential diagnosis of postoperative patients presenting with abdominal symptoms, even years after surgery. It underscores the need for meticulous surgical practices and the role of advanced imaging in timely diagnosis. The successful management of this case demonstrates the critical role of early surgical intervention in preventing morbidity and mortality associated with retained surgical items [1,4,13].

## 4 Conclusion

Gossypiboma, though rare, remains a significant complication following surgical procedures, especially in abdominal and pelvic surgeries. It can present with nonspecific symptoms, often leading to delayed diagnosis and potential morbidity. In this case, a retained surgical gauze migrated into the ileum, presenting as an acute abdominal obstruction with acute appendicitis. Early suspicion, prompt surgical intervention, and thorough postoperative follow-up were key to the patient's favorable outcome. While it is challenging to eliminate the risk of gossypiboma, adherence to meticulous surgical techniques, including accurate sponge counts and intraoperative vigilance, can significantly reduce its occurrence. This case underscores the importance of considering retained foreign bodies in patients with a history of abdominal surgery who present with unexplained abdominal symptoms.

## Consent

Written informed consent was taken from the patient who participated in this study for the publication of this case report and accompanying images.

## Ethical approval

The IRB at our institution has waived ethical approval for case reports.

## Guarantor

The guarantor is Samrat Shrestha.

## Funding

No sources of funding for this case study to declare.

## Author contribution

Constructing hypothesis for the manuscript- Samrat Shrestha, Kaushal S. Thapa, Mecklina Shrestha.

Planning methodology to reach the conclusion: Samrat Shrestha, Kaushal S. Thapa.

Organizing and supervising the course of the article and taking responsibility: Samrat Shrestha.

Patient follow-up and reporting – Kaushal S. Thapa, Mecklina Shrestha.

Logical interpretation and presentation of the results- Samrat Shrestha, Kaushal S. Thapa, Mecklina Shrestha.

Construction of the whole or body of the manuscript- Samrat Shrestha, Kaushal S. Thapa, Mecklina Shrestha.

Reviewing the article before submission not only for spelling and grammar but also for its intellectual content- Samrat Shrestha, Kaushal S. Thapa, Mecklina Shrestha.

## Conflict of interest statement

The authors have no conflict of interest to declare.

## Appendix A Supplementary data

Video 1 Intraoperative video demonstrating the extraction of intraluminal surgical sponge/tetra from ileum. Video 1
